# Scientific Evidence of Rice By-Products for Cancer Prevention: Chemopreventive Properties of Waste Products from Rice Milling on Carcinogenesis* In Vitro *and* In Vivo*

**DOI:** 10.1155/2017/9017902

**Published:** 2017-01-22

**Authors:** Bee Ling Tan, Mohd Esa Norhaizan

**Affiliations:** ^1^Department of Nutrition and Dietetics, Faculty of Medicine and Health Sciences, Universiti Putra Malaysia, 43400 Serdang, Selangor, Malaysia; ^2^Research Centre of Excellent, Nutrition and Non-Communicable Diseases (NNCD), Faculty of Medicine and Health Sciences, Universiti Putra Malaysia, 43400 Serdang, Selangor, Malaysia; ^3^Laboratory of Molecular Biomedicine, Institute of Bioscience, Universiti Putra Malaysia, 43400 Serdang, Selangor, Malaysia

## Abstract

Cancer is a significant global health concern affecting men and women worldwide. Although current chemopreventive drugs could inhibit the growth of cancer cells, they exert many adverse side effects. Dietary factor plays a crucial role in the management of cancers and has drawn the attention of researchers to be used as an option to combat this disease. Both* in vitro* and* in vivo* studies showed that rice and its by-products display encouraging results in the prevention of this disease. The mechanism of anticancer effect is suggested partly through potentiation of bioactive compounds like vitamin E, phytic acid, *γ*-aminobutyric acid (GABA), *γ*-oryzanol, and phenolics. Nevertheless, the bioactivity of rice and its by-products is still incompletely understood. In this review, we present the findings from a preclinical study both in* in vitro* and in animal experiments on the promising role of rice by-products with focus on cancer prevention.

## 1. Introduction

Cancer is a significant health concern. One in eight deaths worldwide is due to cancer [1]. It represents the first or second cause of death in advanced countries. Therefore, urgent action is warranted to reduce the threats of this disease, particularly in developing countries in which the prevalence and incidence of this disease are expected to increase. Genetic defects only account for nearly 5–10% of all cancer cases, whereas 90–95% are due to the environment and lifestyle [2]. Therefore, most of the cancer cases and deaths worldwide are actually preventable [3]. It has also been reported that up to 30% of human cancers could be prevented via an appropriate dietary modification [4].

Carcinogenesis is a complex multistage process comprised of initiation, promotion, and progression stage. Cancer chemoprevention involves the use of natural, synthetic, or biological agents to delay, inhibit, or prevent the initial phases of carcinogenesis, as well as the progression of premalignant cells to invasive disease [5]. In chemoprevention, a vital goal is to block tumor progression [6]. In developed countries, chemopreventive drugs, such as celecoxib and tamoxifen, are being used in high-risk populations; however, due to their adverse side effects, these drugs are not feasible in the developing countries [7–9].

Extensive studies in the past few decades have shown that there are varieties of dietary and botanical natural compounds with chemopreventive properties including garlic, green tea, soy, apple, olive oil, and grape [11–13]. Several recent studies also reported that natural products play a critical role against cancer [14–16]. Identification of bioactive compounds which have medicinal properties from natural products or by-products may promote the nutraceuticals as an inexpensive alternative to anticancer drug therapies which are toxic, immune-suppressive, mutagenic, and even carcinogenic [17, 18]; besides, major treatment modalities include surgery, immunotherapy, and radiotherapy [19–21]. Natural products and their compounds may act as a modulator of antitumorigenicity either as separate entities or by serving in synergism [22]. The combination of two or more components in drug design has also been proven to have clinically synergistic benefits against cancer treatment [23].

In this review, we present the findings from a preclinical study both* in vitro* and* in vivo* on the promising role of rice by-products with focus on cancer perspective.

## 2. Production of Rice By-Products

High demands of crop production in the world are primarily due to the three key forces which are increasing human population, meat and dairy consumption from growing affluence, and biofuel consumption [24–27]. Thus, global agricultural production is projected to increase by 60–110% by 2050 to meet the increasing demands [26, 28, 29] and provide food security to nearly 870 million chronically undernourished individuals [30].

Rice areas with doubling yield rates are only observed in several local areas within Afghanistan, India, Bangladesh, Laos, Vietnam, and Cambodia. In contrast, a significant reduction in the rates of rice yield is reported in some parts of India (particularly in Uttar Pradesh, Maharashtra, and Tamil Nadu) and in North Korea. Rice provides approximately 30% and 27% as a source of energy in India and China, respectively. The world's third largest rice producer, Indonesia, produces about 49% of energy, in which the yield improvement rates are slightly lower at 0.4% per year [32]. The production, area harvested, and yield of paddy in several countries are shown in Table 1.

**Figure 1:**
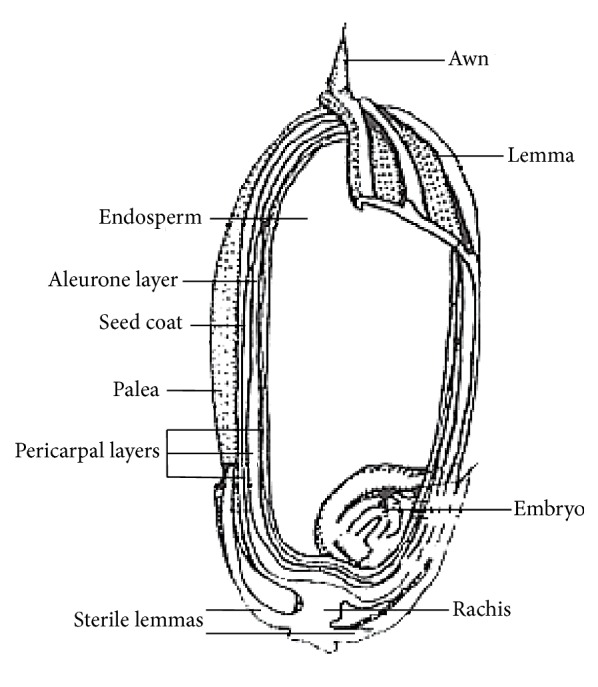
Structure of rice grain [31].

Rice (*Oryza sativa*) is a staple food of dietary calories for half of the humanity and has been widely demonstrated as a chemopreventive component [33]. The rice milling process is comprised of two basic stages: first is to remove the husk to produce brown rice and the other is to remove the bran layer from brown rice to produce the polished (or white) rice. Additionally, the milling process also separates the germ and some of the endosperm as broken kernels and powdery materials [34]. Therefore, the output of a rice milling process consists of a main product, which is milled rice, and several rice by-products such as the husk, germ, bran layer, and broken kernels [35]. Traditionally, rice is consumed as polished white rice with the removal of husk, bran, and germ fractions [36], but currently the consumption of brown rice or germinated brown rice is getting popular due to more people becoming aware about their health. The production of rice by-product in rice processing was previously described by Esa et al. [37]. Structure of rice grain is shown in Figure 1.

## 3. Nutrient Composition and Phytonutrients Properties of Rice By-Products

High intake of whole grain food has been suggested as an indicator of the reduction of risk in several digestive tract neoplasms, such as stomach, colon, and gall bladder [59]. In an earlier study, Deng et al. [60] studied the health beneficial components in pigmented rice such as flavones, tannins, phenolics, sterols, tocols, *γ*-oryzanols, amino acids, *γ*-aminobutyric acid (GABA), and essential oils. These compounds have been shown to have a variety of bioactivities including antitumor, antioxidant, antiatherosclerosis, hypoglycemic, and antiallergic activities. The other components in whole grain such as indigestible fibers (Tables 2 and 3), as well as other phytochemicals as illustrated in Tables 4 and 5, have also been considered to contribute to the beneficial effects of whole grain [61].

Rice bran has been extensively studied among all rice by-products. Besides containing high amounts of vitamins, minerals, and fiber, rice bran also is rich in numerous bioactive components which draw a great deal of attention in the prevention and treatment of several types of human cancers. These bioactive components were reported to have antioxidant activities that can be directly associated with their anticancer effects [62] including leukemia, breast, lung, liver, cervical, stomach, and colorectal cancers [33, 63]. Studies by Moldenhauer et al. [64], Renuka Devi and Arumughan [65], and Canan et al. [66] further supported the role of a unique complex of naturally antioxidant compounds in rice bran to fight several diseases including cancer.

In addition to the rice bran, more than 1 million tons of rice husks is produced annually in rice processing in South Korea. Rice husk consists of 20% of the rice grain kernel and is nearly similar to other plant biomasses, containing a high proportion of organic substances [75]. Rice husk is inedible, which is utilized in many nonfood applications as low-value agricultural waste product. However, it was reported that rice husk is a valuable source of bioactive compounds that contained a high antioxidant property. Rice husk contained phenolic acids, which are a valuable source of natural antioxidants and prevent the rice seed from oxidative stress [70]. Thus, it has been recognized as a potential source of energy and organic chemicals [76, 77]. Hydrothermal treatments of rice husks also produce lignin-derived components such as caffeic acid and ferulic acid [78], which are crucial constituents for pharmaceutical application because they provide protection against photooxidative damage [79]. Rice husk has also been reported to have fatty acids, including linoleic, stearic, oleic, and palmitic acids [78]. Phenolic compounds in the methanolic extract of rice husk also displayed a high antioxidant activity against scavengers of singlet oxygen and suppressed high hydrogen peroxide-induced damage against cellular deoxynucleic acid (DNA) in human lymphocytes [80].

Rice germ is also a by-product from rice processing which is known as embryo or reproductive proportions which germinate and grow into a plant [57]. The amount of vitamin E in rice germ is 5 times higher compared with that of rice bran. Most of the vitamin E in rice germ is *α*-tocopherol, which is the major active form of vitamin E, while, for rice bran, most of the vitamin E is *γ*-tocopherol. Additionally, rice germ also comprised a substantial amount of vitamins (B_1_, B_2_, and B_6_), dietary fiber, and neurotransmitter GABA, which is believed to contain numerous beneficial health effects like improving blood pressure, cognition, and blood glucose levels. The amount of *γ*-oryzanol in rice germ, however, was 5 times lower than that of rice bran [81].

In addition to the rice bran, rice husk, rice straw, and rice germ, brewers' rice is another by-product in the rice industry that has a significant nutritional value. Brewers' rice consists of a mixture of broken kernels with rice bran and rice germ, which is usually removed during the rice milling process [82]. Brewers' rice is typically used as an animal feed and brewing material [83]. The size of brewers' rice is less than one quarter of the full kernel of milled rice. It is the last and the smallest milling portion that is separated during the milling of paddy rice and is usually removed from larger rice kernels [84]. Brewers' rice demonstrated high carbohydrate, protein, and fat contents [55]; minerals such as calcium, phosphorus, iron, sodium, and potassium; fatty acids such as linoleic acid, oleic acid, and palmitic acid. Brewers' rice also contained a variety of phytochemicals with chemopreventive properties including *γ*-oryzanol, phytic acid, vitamin E, phenolic antioxidants [72], and dietary fiber [55]. Compared with other cereal brans, such as corn, wheat, and oat, the lipid proportion present in brewers' rice has a unique ratio of vitamin E isoforms (*α*-, *γ*-, and *δ*-tocopherols and tocotrienols) and *γ*-oryzanol [72].

Since phenolic compounds are demonstrated to confer beneficial health benefits, this may partially explain a better nutritional value of rice by-products. Biological activities in the cereal grains were strongly correlated to their polyphenols content [85], which is known to exhibit potent antioxidant activities [86–88]. Majority of the antioxidants commonly present in fruits, vegetables, and cereals (wheat, rice, and oat) are polyphenolic compounds [89–92]. Li et al. [89] reported that most of the phenolic acids present in the whole grains are ferulic acid, p-coumaric acid, vanillic acid, caffeic acid, and syringic acid. Others have also reached a similar finding, in which the rice grains were rich in ferulic acid and p-coumaric acid [93, 94]. In view of the total phenolic content in various genotypes of rice by-products, rice brans have 2–11-fold higher content than rice husk, 4–15-fold higher content than brown rice, and 7–59-fold greater content than polished rice [95].

## 4. Anticancer Activities of Rice By-Products

### 4.1. *In Vitro* Cancer Chemopreventive Study

Colorectal cancer chemoprevention activity highlighted the role of bioactive constituents like rice bran phytic acid [96], tricin, and flavonoids [97]. Relative proportions of bioactive components in rice bran have been shown to inhibit the growth of colorectal cancer cells; however, they are different among other rice varieties [71]. Kong et al. [98] reported that rice bran phenolic compound cycloartenyl ferulate inhibited the proliferation of human colorectal adenocarcinoma (SW480) cell line. Chen et al. [63] used different cancer cell lines to study the cell-inhibiting activity in response to red rice bran extract and demonstrated that red bran exhibited strong inhibitory effects against leukemia, cervical, and stomach cancers. Forster et al. [71] also reported that total phenolics and *γ*-tocotrienol from rice bran exhibited significantly reduced colorectal cancer cell proliferation (*p* < 0.05). Another rice by-product, momilactone B, an allelochemical of rice hull, has antiproliferative activity against human leukemic T-cells via activation of caspase and mitochondria pathways [99]. In addition to the effects observed in leukemic cancer, methanol extract of rice husk also inhibited the growth of colon cancer cells with inhibition concentration (IC_50_) values of 0.5 *μ*g/mL [100].

Study on the effect of brewers' rice on colorectal cancer (HT-29) cell line was conducted by Tan et al. [72, 74]. They reported that water extract of brewers' rice (WBR) inhibited the proliferation of HT-29 cell line and the effect was suggested to be linked to the bioactive compounds present in WBR. The extract, however, was not cytotoxic against normal cell lines [72]. This finding was consistent with the data obtained by Ryan et al. [101], Fan et al. [102], and Kong et al. [98], who reported that the rice bran components were not cytotoxic against normal cell lines. Summary of* in vitro* studies on antiproliferative effect of rice by-products is shown in Table 6.

### 4.2. *In Vivo* Cancer Chemopreventive Study

Rice germ or the constituents present in the rice bran or germ have been identified to have chemopreventive effects against carcinogenesis in the colon [125], liver [126], stomach [127], esophagus [128], and bladder [129] of rodents. Kong et al. [98] observed that rice bran cycloartenyl ferulate significantly induced suppression of human colorectal adenocarcinoma (SW480) of xenograft in nude mice after 21 days and triggered both death receptor and mitochondrial apoptosis pathways. Similarly, Kim et al. [122] used pathogen-free female BALB/c mice to evaluate the effect of rice bran *γ*-oryzanol on colon tumor growth and found that rice bran *γ*-oryzanol could inhibit colon tumor and reduce expression of vascular endothelial growth factor (VEGF), cyclooxygenase-2 (COX-2), and 5-lipoxygenase (5-LOX). In another study, Choi et al. [123] further demonstrated that feeding a diet containing 10% (w/w) black and brown rice brans reduced VEGF, COX-2, and 5-LOX expression in mouse colon carcinoma cells- (CT-26-) treated mice. Increased COX-2 expression, an inflammatory gene, is positively associated with the inflammatory response strength [130]. Reduction of COX-2 suggests that black and brown rice brans could attenuate the inflammatory response in cancer through reduction of oxidative stress. Interestingly, feeding with rice bran not only reduced the number of aberrant crypt foci (ACF) [120] but also improved lipid profile as described by Ausman et al. [131] suggesting the numerous functional potentials of rice bran. Additionally, Kawabata et al. [113] reported that feeding azoxymethane (AOM) (15 mg/kg body weight once weekly for 3 weeks) with rice germ for 5 weeks significantly suppressed colon adenocarcinoma (*p* < 0.01) in rats. Mori et al. [114] also observed that the ACF/colon in the rats induced with AOM and fed with rice germ (2.5% in diet) were significantly reduced compared to those of the group with AOM only (*p* < 0.005). In addition to the effects observed on rice bran and rice germ, using male F344 rats model that received carcinogen 1,2-methylhydrazine (DMH) subcutaneously once weekly for 6 weeks at a dosage of 180 mg/kg body weight, Kim et al. demonstrated that the methanolic extract of rice husk could reduce colon preneoplastic ACF formation by 35% after 40 weeks (*p* < 0.01) [100]. In view of the apoptotic-inducing efficacy observed in HT-29 cell line on water extract of brewers' rice (WBR), findings from an animal study mirror some of those from preclinical data obtained from an* in vitro* study. Tan et al. [55] reported that feeding AOM-induced colon cancer rats (15 mg/kg body weight) with a diet containing 40% brewers' rice reduced significantly colon tumor multiplicity after 20 weeks of treatment (*p* < 0.05). The suppressive effects seen in the highest concentration of brewers' rice treatment could be explained by its higher concentrations of active compounds that may confer better functional properties. However, it is not yet clearly understood which bioactive constituents are responsible for the functional benefits of brewers' rice, but it is more likely that several of the phytonutrients contribute towards these observed effects. Among the studies described to date, the improvements in these indices could be attributed to higher levels of *γ*-oryzanol, phytic acid, vitamin E, and antioxidants [72]. So, it is important to encourage long-term clinical studies to verify these findings by providing a better alternative to curb the rising incidence and prevalence of colon cancer.

Several studies as reported by Henderson et al. [33] also demonstrated that rice by-products have chemopreventive potential not only in the colon but also in the breast, lung, mouth, bladder, liver, esophagus, and melanoma/skin* in vivo*. Wang et al. [119] observed that defatted rice bran sulfated polysaccharide (SRBPS2a) suppressed the implanted EMT-6 breast tumor cells growth in BALB/c mice. In another study, Yasukawa et al. [112] demonstrated that feeding ICR mice with rice bran cycloartenol ferulate is possible to suppress tumor promotion in 2-stage skin carcinoma. Furthermore, defatted rice germ has also been reported to reduce the incidence of tongue carcinomas and preneoplastic lesions in Fisher 344 rats [114] (Table 7).

The beneficial effects of bioactive components observed in rice by-products, as reported above, have been postulated by the concept of food synergy [132]. If so, the synergistic/additive effects of bioactive compounds in rice by-products could in the long term be beneficial in the management of cancer via multiple mechanisms perhaps even better than some drugs as suggested by Ricciardiello et al. [133] who suggested that whole food or whole food extract can have high importance in combating carcinogenesis. These observations also supported the finding by Tsuda et al. [134] who demonstrated a synergistic and/or additive protective effect of several bioactive compounds.

## 5. Mechanisms of Action of Rice By-Products as Anticancer Agent

### 5.1. Apoptosis Induction and Inhibition of Cancer Cellular Proliferation

Apoptosis or programmed cell death plays a crucial role in the tissues maintenance, organ homeostasis, and genetically controlled cell death to balance cell proliferation [135]. Apoptosis is a normal and continuous process in healthy subjects accompanied with complex physiological processes, which controls some of the vital cellular processes like cell turnover, maintaining homeostasis of cell population, development of the immune system, hormone-dependent atrophy, embryonic development, and chemical-induced cell death [136]. Inappropriate regulation of apoptotic cell death mechanism has been identified in numerous human diseases. Thus, aberrant apoptosis contributes to cancer progression. Apoptosis can be stimulated via internal and external stimuli. An internal stimulus can be a* p53 *tumor suppressor gene, while external stimulus is external plasma membrane associated receptors [137].

The ability to modulate cell death is identified as a potential therapeutic agent for cancer. Numerous bioactive compounds present in rice bran like ferulic acid [95], *γ*-oryzanol, phytic acid [138], p-coumaric acid [139], pectin [140], tricin, and tocotrienol-tocopherols are believed to be responsible for inducing apoptosis [101]. As shown by Serafim et al. [141], caffeic and ferulic acid derivatives play a crucial role in the increase of tumor suppressor protein p53 expression and enhance the mitochondrial depolarization and chromatin condensation. In addition to the effects observed on caffeic and ferulic acid, vitamin E, particularly tocotrienols, has also been shown to induce cell cycle arrest [142], activate p53 and caspase activity [143, 144], inhibit adhesion molecules [145], suppress nuclear factor-kappa B (NF-*κ*B) [146], and downregulate c-Myc and telomerase [147]. Kannappan et al. [148] further demonstrated that *γ*-tocotrienol not only has the ability to downregulate the Bcl-2 and Bcl-xL antiapoptotic proteins expression but also induces SHP-1 expression, which directly suppresses the activation of STAT3, as STAT3 pathway correlated well with cancer progression. Furthermore, *γ*-tocotrienol-induced apoptosis was also demonstrated in Hep3B cells via caspase-3, caspase-8, and caspase-9 activities with the participation of Bax and Bid [149]. Findings by Ahn et al. [146] further showed that *γ*-tocotrienol suppressed the NF-*κ*B activation pathway via inhibition of receptor-interacting protein (RIP) and TAK1 and thus suppressed the antiapoptotic gene and contributed to apoptosis. Another vitamin E isomer, *δ*-tocotrienol, also induced apoptosis in human breast cancer cells through involvement of transforming growth factor-*β*, Fas, and c-Jun N-terminal kinase (JNK) signaling pathways [150].

In addition to the bioactive compounds mentioned above, *β*-sitosterol has also been reported to have a beneficial effect against colorectal, stomach, and breast cancers [151, 152]. The possible mechanism is by increasing the damage of the cancer cell membrane, activation of caspase-3 activity [152], and increasing the production of cellular ceramide, which is related to the cell cycle arrest [151]. Study on the effect of water extract of brewers' rice (WBR) treatment also resulted in the induction of apoptosis by significant activation of caspase-3 and caspase-8 activities [153]. Momilactone B, an allelochemical extracted from rice husks, was also shown to induce apoptosis in human lymphoma (Jurkat) cells through caspase and mitochondria pathways [99].

In addition to the prevention of the initiation stage of cancer via the induction of apoptosis, it is also vital for cancer chemopreventive agents to suppress the proliferation of malignant cells. Phytic acid is generally known as an antinutrient compound due to its propensity to form a complex with minerals and subsequently contributed to deficiencies in animal/human. It was also revealed from several* in vitro* and* in vivo* studies to have potential in suppressing abnormal cell proliferation [124, 154].

The efficacy of these bioactive compounds as anticancer agents, however, depends on the bioavailability and the dosage [155]. For example, gastrointestinal esterases in the large and small intestines of humans and rats can release diferulic acid from bran fiber, which may promote its bioavailability [156]. In addition, ferulic acid also remains in the bloodstream longer and hence may confer more protection than other known antioxidants. On the other hand, even though phytic acid has been shown to inhibit the proliferation of cancer cells, the absorption in the small intestines of humans is low and relies on plasma concentration [157, 158]. However, several studies suggested that the bioavailability of phytic acid depends on the source of food that it comes from.

### 5.2. Regulation of Wnt/*β*-Catenin Signaling Pathway

**Figure 2:**
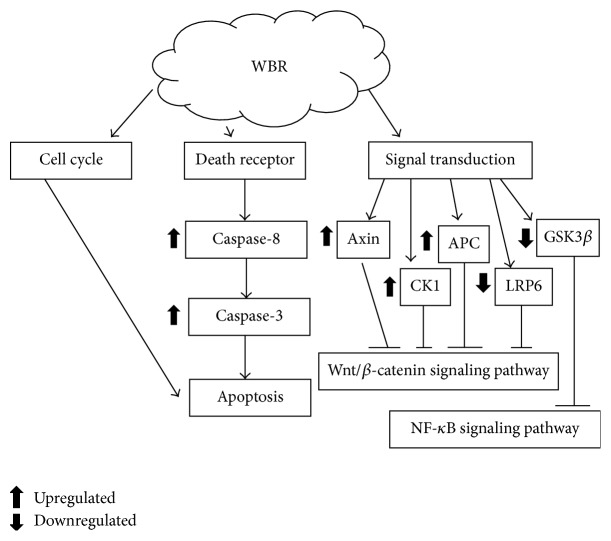
Summary of mechanisms involved in colorectal cancer (HT-29) cells of water extract of brewers' rice (WBR). APC: adenomatous polyposis coli; CK1: casein kinase 1; GSK3*β*: glycogen synthase kinase 3*β*; NF-*κ*B: nuclear factor-kappa B; LRP6: low-density lipoprotein receptor-related protein 6; WBR: water extract of brewers' rice.

**Figure 3:**
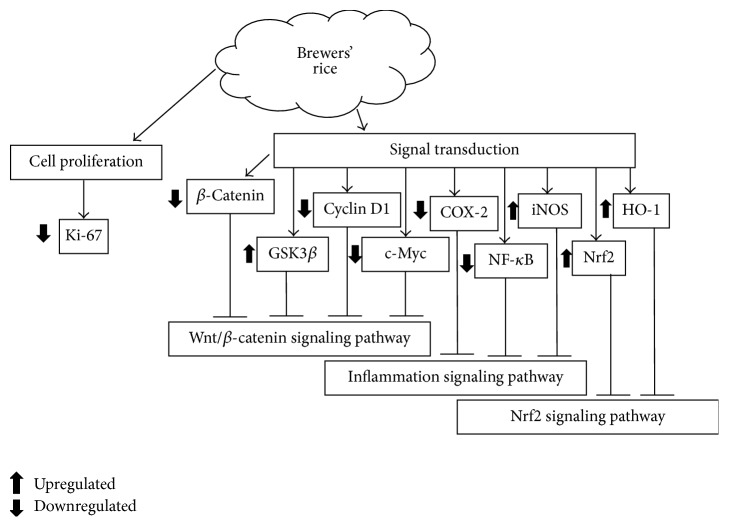
Summary of mechanisms involved in colon tumorigenesis of brewers' rice. COX-2: cyclooxygenase-2; GSK3*β*: glycogen synthase kinase 3*β*; HO-1: heme oxygenase-1; iNOS: inducible nitric oxide synthase; NF-*κ*B: nuclear factor-kappa B; Nrf2: NF-E2-related factor 2.

Deregulation of Wnt/*β*-catenin signaling has been demonstrated to be associated with cancer especially with colorectal cancer [159–164]. Cyclin D1 is a well-known cell cycle protein targeted by *β*-catenin [165] that is frequently overexpressed in colon tumor tissues [166]. c-Myc is another vital protein in the regulation of cell growth by *β*-catenin and the Wnt pathway [167]. Leardkamolkarn et al. [168] reported that methanol extract of Thai rice, Riceberry bran, diminished the amount of cyclin D1 in colonic carcinoma (Caco-2) cell line. In this study, reduction of cyclin D1 observed in colon carcinoma cells, however, was attributed to vitamin E or tocotrienol content [169]. Similarly, Sun et al. [170] and Gysin et al. [169] reported that vitamin E or tocotrienol reduced the expression of cyclin D1 in gastric adenocarcinoma and prostate carcinoma. In addition to vitamin E, rice bran phytic acid has also been reported to have a similar effect in diminishing the expression of *β*-catenin, which could potentially reduce colon carcinogenesis [171]. Inhibition of colon carcinogenesis via modulation of Wnt/*β*-catenin signaling pathway was also shown in brewers' rice and its water extract (WBR). As indicated in Figure 2, WBR upregulated mRNA levels of casein kinase 1 (*CK1*) and adenomatous polyposis coli (*APC*), a destruction complex involved in the degradation of *β*-catenin, and inhibited the low-density lipoprotein receptor-related protein 6 (*LRP6*), which is a crucial coreceptor in Wnt signaling, and glycogen synthase kinase 3*β* (*GSK3β*) mRNA levels [153]. The fact that WBR downregulates* GSK3β* and produces better colon tumor inhibition suggests that other mechanisms are involved in WBR's anti-colon cancer properties and are likely modulated by NF-*κ*B [172, 173]. Additionally, brewers' rice also reduced *β*-catenin, cyclin D1, and c-Myc expression, as illustrated in Figure 3 [153]. Preclinical findings from* in vitro* and* in vivo* studies on mechanisms involved in anti-colon cancer effects suggest that rice by-products could modulate Wnt pathway in colon cancer.

### 5.3. Modulation of Inflammation Pathway

Numerous bioactive components present in rice by-products have been demonstrated to facilitate cancer chemoprevention through the enhancement of immune response. These cancer chemopreventive immune responses serve either via blocking viral infections related to virus-induced tumors or by specific suppression of tumor cells [174]. The early stages of an inflammatory response are vital in the protection against infection and injury; however, an uncontrolled or chronic inflammatory environment is favorable towards cancer development [33]. Chronic infection and inflammation stimulate the inflammatory-associated genes such as NF-*κ*B [175] and inducible nitric oxide synthase (*iNOS*) expression [176]. As reported by Henderson et al. [177], the whole dietary rice bran modulates the mucosal immune response by increasing the numbers of mesenteric lymph nodes and lamina propria dendritic cells [177]. They also revealed that ferulic acid from rice bran has potential to be a promising cancer chemopreventive agent to inhibit the lipopolysaccharide- (LPS-) induced iNOS and COX-2 protein expression as well as suppress the release of tumor necrosis factor-*α* (TNF-*α*). This phenomenon was associated with the inhibition of I-*κ*B phosphorylation and subsequently suppression of NF-*κ*B signaling [178].

An earlier study by Morel et al. [179] reported the ability of *α*-tocopherol to promote cytokine production and monocyte recruitment, which are required in the adaptive immunity development. Boxer [180] and Sakurai et al. [181] also showed that *α*-tocopherol and ferulic acid promote the respiratory burst and interferon-*γ* production in macrophages, respectively. A previous study by Sierra et al. [182] also reported the ability of rice bran oil to regulate the immune response by promoting the proliferation of B-cell and inducing interleukin-2 (IL-2) and TNF-*α* production. In addition, rice bran extract MGN-3 has been demonstrated to promote dendritic cell maturation [183] and enhance cytokine production and natural killer (NK) cell activity [184].

Feeding mice with diet supplemented with black rice bran extract prior to 12-O-tetradecanoylphorbol-13-acetate (TPA) administration was also shown to inhibit inflammation (edema) caused by TPA by a marked reduction in proinflammatory cytokines TNF-*α*, interleukin-1*β* (IL-1*β*), interleukin-6 (IL-6), and eicosanoids leukotriene B4 (LTB4) [185]. Gamma-oryzanol primarily consists of esters of trans-ferulic acid (trans-hydroxycinnamic acid) and phytosterols (sterols and triterpenic alcohols). Cycloartenol, *β*-sitosterol, 24-methylenecycloartenol, and campesterol are present predominantly in *γ*-oryzanol [186, 187]. Anti-inflammatory effects of *γ*-oryzanol and cycloartenyl trans-ferulate markedly suppressed the inflammatory response in mice-induced colitis [188]. Accordingly, Islam et al. [189] demonstrated that rice bran phytosteryl ferulates modulated anti-inflammatory reactions via downregulation of inflammatory transcription factor, NF-*κ*B, which may lead to the reduction of inflammatory enzymes like COX-2 and iNOS and proinflammatory cytokines such as IL-1*β*, IL-6, and TNF-*α*. Rice bran phytosteryl ferulates further enhanced blood adiponectin levels through indirect stimulation of peroxisomal proliferator-activated receptor-*γ* (PPAR*γ*) via suppression of NF-*κ*B. Collectively, rice bran *γ*-oryzanol inhibited tumor growth via stimulation of cytolytic activity in splenic (NK) cells, partial restoration of nitric oxide production, and phagocytosis in peritoneal macrophages, subsequently resulting in the liberation of proinflammatory cytokines TNF-*α*, IL-1*β*, and IL-6 from macrophages.

MGN-3/biobran, arabinoxylan rice bran, was shown to have antitumor activity, through modulation of immune systems such as NK cells [190–192], TNF-*α* [184, 193], and macrophages [194]. The investigators found that MGN-3 suppresses tumor in solid Ehrlich carcinoma- (SEC-) bearing mice via induction of apoptosis through its immunomodulatory effects [184]. Additionally, isovitexin from rice husk has also been demonstrated to inhibit release of COX-2 expression and reduced LPS-stimulated prostaglandin E_2_ [195]. In addition to the effects observed in rice bran and rice husk in the inflammation pathway, brewers' rice has also been demonstrated to have a similar effect against COX-2 activity. It was shown to reduce COX-2 and NF-*κ*B levels. Findings from animal study also revealed that brewers' rice upregulated* iNOS* [196]. This is in agreement with an earlier finding that overexpression of iNOS attenuates the proliferation and metastasis of human renal cell carcinomas and murine fibrosarcoma [197]. In this regard, natural components present in the brewers' rice such as polyphenolic compounds, including ferulic acid, gallic acid, p-coumaric acid, syringic acid, vanillic acid [74], and vitamin E isomers, *γ*-oryzanol [72], and *γ*-aminobutyric acid (GABA) [198] have the potential to inhibit the proinflammatory immune signaling and subsequently reduce the colon cancer development.

### 5.4. Protection against Free Radicals and Modulation of Antioxidant Pathway

One of the effective mechanisms for cancer prevention is to inhibit initiation stage through suppression of the DNA damage caused by reactive oxygen species (ROS) or other carcinogens [199]. Oxidative stress can cause significant cellular damage and irreversible mutations; thus, a substantial amount of antioxidant may protect cells from free radical damage.

Study by Tan et al. [196] showed that dietary administration of brewers' rice helped to protect against oxidative stress in AOM-induced rat colon carcinogenesis by improved antioxidant capacity like superoxide dismutase (SOD), malondialdehyde (MDA), and nitric oxide (NO). They also observed that brewers' rice upregulated NF-E2-related factor 2 (Nrf2) signaling pathway via modulation of Nrf2 and heme oxygenase-1 (HO-1) transcriptional activities (Figure 3). Earlier study by Tan et al. [55] also reported that brewers' rice consists of phenolic antioxidants, phytic acid, vitamin E, oryzanol, and *γ*-aminobutyric acid (GABA) which were shown by many previous studies to have a good antioxidant capacity. Ferulic acid, for example, is a well-documented phenolic compound that can be found in high amount in rice by-products, demonstrated to be an effective superoxide anion radical scavenger and lipid peroxidation inhibitor [155]. In another study, ferulic acid has been reported to protect against hydrogen peroxide-induced cellular damage via elevation of cellular levels of HO-1 and heat shock protein-70 [200]. Overall, this suggests that the protective effect of rice by-product on oxidative stress may be mediated partly via the synergistic/additive effects of these bioactive constituents.

Besides bioactive compounds stated above, MGN-3, an arabinoxylan extracted from rice bran, has also been reported to induce oncostatic activity against murine solid Ehrlich carcinoma via modulation of lipid peroxidation and enhanced the endogenous antioxidant scavenging activity such as SOD, glutathione peroxidase (GPx), catalase (CAT), and glutathione-S-transferase (GST) [201].

## 6. Summary and Future Prospects

This review has provided substantial evidence both* in vitro* and* in vivo* that consumption of rice by-products may provide the optimal chemoprevention due to antioxidant phytonutrients. Further clinical studies of rice by-products and their unique bioactive compounds hold great promise in future use as a dietary cancer chemopreventive agent.

In conclusion, rice by-products as a cancer chemopreventive dietary agent represent a unique approach to evaluate effective whole food compared to the individual phytochemical. Our review showed a promising result from both* in vitro* and* in vivo* studies, which warrants clinical studies designed to gain a better understanding of the relationship between rice bran/rice husk/rice germ/brewers' rice and cancers. The global availability and affordability of rice by-products provide a better public health opportunity in both developed and developing countries. Taken together, this review could pave the way for the potential use of rice by-products as a functional food in the prevention and treatment of cancers. The potential implication of the dietary intake of rice by-products in place of conventional treatment modalities could be significant and is warranted to be evaluated further in long-term clinical studies.

## Figures and Tables

**Table 1:** Production, area harvested, and yield of paddy [10].

	2013			2003		
Regions	Area harvested (Ha)	Production quantity (tons)	Yield (Hg/Ha)	Area harvested (Ha)	Production quantity (tons)	Yield (Hg/Ha)
China	30486000	205015000	67249	26780124	162304280	60606
India	43500000	159200000	36598	42592500	132789000	31177
Indonesia	13835252	71279709	51520	11477357	52137600	45426
Thailand	12373163	38787697	31348	10163878	29473521	28998
Vietnam	7902808	44039291	55726	7452200	34568800	46387
Malaysia	688207	2626881	38170	671800	2257000	33596

Ha: hectares; Hg/Ha: hectogram/hectare.

**Table 2:** Proximate composition and minerals contents (% of dry matter) of rice by-products.

Nutrients	Broken^a^	Husk^b^	Bran^c^	Polishings^d^	Straw^e^	Brewers' rice^f^
Dry matter	87.0–89.0	87.0–92.5	89–94	90.0	90.9	—
Protein^g^	6.7–9.8	2.1–4.3	10.6–16.9	11.2–13.4	1.2–7.5	9.01 ± 0.27
Crude fat	0.5–1.9	0.30–0.93	5.1–19.7	10.1–13.9	0.8–2.1	1.95 ± 0.11
Crude fiber	0.6	30.0–53.4	7.0–18.9	2.3–3.6	33.5–68.9	—
Ash	5.0	13.2–24.4	8.8–28.8	5.2–8.3	12.2–21.4	1.56 ± 0.26
Carbohydrate	—	22.4–35.3	90.0	51.1–55.0	39.1–47.3	72.42 ± 1.25
Calcium	0.09–0.19	0.04–0.21	0.08–1.4	0.05	0.30–0.71	0.013 ± 0.0016
Phosphorus	0.03–0.04	0.07–0.08	1.3–2.9	1.48	0.06–0.16	0.316 ± 0.028

^a^[38–42].

^b^[38, 39, 43–48].

^c^[38–40, 42–50].

^d^[41, 42, 48].

^e^[44, 45, 51–54].

^f^[55].

^g^Animal scientists commonly use a conversion factor of *N* × 6.25 for crude protein [56].

**Table 3:** Dietary fiber in rice husk, rice bran fiber, and rice straw.

	Rice husk^a^	Rice bran fiber^a^	Rice straw^b^
Cellulose (%)	38.0	30.0	32.0
Hemicellulose (%)	20.0	20.0	35.7
Lignin (%)	22.0	20.0	22.3

^a^[57].

^b^[58].

**Table 4:** Phytochemicals and antioxidant properties of rice by-products.

Phytochemicals or antioxidant properties	Rice bran	Rice husk	Brewers' rice
Gamma-oryzanol (mg/g)	Methanol extract of defatted rice bran (India) = 7.82^a^	Acetone extract of Thai rice husk variety (Khao Dawk Mali 105) = 0.06–0.16^e^	14.20^h^
	Methanol extract of rice bran from Thailand = 0.56–1.08^b^		
	Ethanol extract of rice bran from Suphan Buri (Thailand) = 9.81^c^		
	Methanol extract of *Japonica *rice bran = 9.8 ± 0.4^d^		
	Ethyl acetate extract of *Japonica *rice bran = 13.8 ± 0.9^d^		
	Hexane extract of *Japonica *rice bran = 13.1 ± 0.5^d^		
	Acetone extract of Thai rice bran variety (Khao Dawk Mali 105) = 3.43–5.38^e^		
	80% methanol extract of whole rice bran = 0.93–5.13^g^		

Tocopherols (*µ*g/g)	Methanol extract of defatted rice bran (India) = 138^a^	Acetone extract of Thai rice husk variety (Khao Dawk Mali 105) = not detected^e^	3.4^h^
	Methanol extract of rice bran from Thailand = 350–670^b^		
	Hexane extract of rice bran from Suphan Buri (Thailand) = 172^c^		
	Methanol extract of *Japonica *rice bran = 573 ± 13^d^		
	Ethyl acetate extract of *Japonica *rice bran = 770 ± 24^d^		
	Hexane extract of *Japonica *rice bran = 196 ± 24^d^		
	Acetone extract of Thai rice bran variety (Khao Dawk Mali 105) = 70.67–87.54^e^		
	80% methanol extract of whole rice bran = 27.4–129.6^g^		

Tocotrienols (*µ*g/g)	Methanol extract of rice bran from Thailand = 220–460^b^	—	3.25^h^
	Methanol extract of *Japonica *rice bran = 202 ± 5^d^		
	Ethyl acetate extract of *Japonica *rice bran = 237 ± 6^d^		
	Hexane extract of *Japonica *rice bran = 17 ± 1^d^		
	80% methanol extract of whole rice bran = 20.8–301.7^g^		

Total phenolic content (g GAE/kg)	Methanol extract of *Japonica *rice bran = 15.7 ± 0.6^d^	—	—
	Ethyl acetate extract of* Japonica *rice bran = 19.7 ± 0.8^d^		
	Hexane extract of *Japonica *rice bran = 14.7 ± 1.2^d^		

Total anthocyanins (mg/g extract)	70% ethanol extract of purple rice bran = 55.7 ± 2.1^f^	—	—

Total proanthocyanidins (mg pro. B2 equiv./g extract)	70% ethanol extract of red rice bran = 66.88 ± 6.23^f^	—	—

Phytic acid (mg/g)	—	—	0.38 ± 0.01^h^

GAE: gallic acid equivalents.

^a^[65].

^b^[67].

^c^[68].

^d^[69].

^e^[70].

^f^[63].

^g^[71].

^h^[72].

**Table 5:** Polyphenolic compounds present in the rice by-products.

Polyphenolic compounds	Rice bran	Rice husk	Brewers' rice
Gallic acid (*µ*g/g)	—	80% methanol extract = 5.4–9.9^a^	Water extract = 26.09 ± 2.01^d^

Protocatechuic acid (*µ*g/g)	70% ethanol extract of light brown rice bran = not detected^c^	80% methanol extract = 6.7–24.0^a^	—
	70% ethanol extract of purple rice bran = 5777 ± 98^c^		
	70% ethanol extract of red rice bran = 168 ± 4^c^		

p-Hydroxybenzoic acid (*µ*g/g)	—	80% methanol extract = 10.8–110.4^a^	—

Chlorogenic acid (*µ*g/g)	—	80% methanol extract = 4.8–11.3^a^	—

Vanillic acid (*µ*g/g)	70% ethanol extract of light brown rice bran = 34 ± 2^c^ 70% ethanol extract of purple rice bran = 1568 ± 36^c^ 70% ethanol extract of red rice bran = 72 ± 3^c^	80% methanol extract = 7.8–14.1^a^ 80% aqueous methanol = 14.4–26.7^b^	Water extract = 2.87 ± 0.15^d^

Syringic acid (*µ*g/g)	—	80% methanol extract = 2.6–12.1^a^	Water extract = 5.87 ± 1.71^d^

p-Coumaric acid (*µ*g/g)	70% ethanol extract of light brown rice bran = 424 ± 1^c^ 70% ethanol extract of purple rice bran = 517 ± 49^c^ 70% ethanol extract of red rice bran = 511 ± 2^c^	80% methanol extract = 14.8–32.5^a^ 80% aqueous methanol = 4.84–28.90^b^	Water extract = 7.13 ± 0.36^d^

Ferulic acid (*µ*g/g)	80% aqueous methanol = 20.70–33.45^b^	80% methanol extract = 18.1–64.2^a^	Water extract = 36.42 ± 2.97^d^
	70% ethanol extract of light brown rice bran = 1995 ± 4^c^		
	70% ethanol extract of purple rice bran = 1593 ± 67^c^		
	70% ethanol extract of red rice bran = 1055 ± 11^c^		

4-Hydroxybenzoic acid (*µ*g/g)	70% ethanol extract of light brown rice bran = 673 ± 10^c^	—	—
	70% ethanol extract of purple rice bran = not detected^c^		
	70% ethanol extract of red rice bran = 427 ± 15^c^		

Caffeic acid (*µ*g/g)	70% ethanol extract of light brown rice bran = 157 ± 14^c^	—	Water extract = 5.32 ± 2.48^d^
	70% ethanol extract of purple rice bran = Not detected^c^		
	70% ethanol extract of red rice bran = 111 ± 3^c^		

Sinapic acid (*µ*g/g)	70% ethanol extract of light brown rice bran = 2544 ± 17^c^	—	—
	70% ethanol extract of purple rice bran = 2039 ± 82^c^		
	70% ethanol extract of red rice bran = 2266 ± 16^c^		

^a^[73].

^b^[70].

^c^[63].

^d^[74].

**Table 6:** Anticancer effect of rice by-products *in vitro*.

Author(s)	Year	Components	Findings
Hudson et al. [62]	2000	Rice bran phenolic extracts (tricin, ferulic acid, caffeic acid, and methoxycinnamic acid)	Reduced the number of viable SW480 cells and inhibited the colony-forming ability.

Luo et al. [103]	2005	Gamma-oryzanol (cycloartenyl trans-ferulate and 24-methylenecycloartanol trans-ferulate) from rice bran	Moderate cytotoxicity effect against MCF-7 cells.

Kim et al. [100]	2007	Methanol extract of rice husk	Highly cytotoxic against colon cancer cells, with IC_50_ value of 0.5 *μ*g/mL.

Gollapudi and Ghoneum [104]	2008	MGN-3/biobran, modified arabinoxylan from rice bran	Treatment with MGN-3 increased susceptibility of human breast cancer cells to daunorubicin (5.5-fold for MCF-7 and 2.5-fold for HCC70 cells) compared with that of human breast cancer cells treated with daunorubicin alone.

Joung et al. [105]	2008	Momilactone B, an allelochemical of rice husk	Suppressed hypoxia-induced increases of cyclin D1 in human breast cancer cells.

Kannan et al. [106]	2008	Peptide hydrolysates derived from heat-stabilized defatted rice bran	Suppressed the proliferation of Caco-2 and HepG2 cancer cells.

Lee et al. [99]	2008	Momilactone B, an allelochemical of rice husks	Inhibited the proliferation of human leukemic T-cells (Jurkat).

Punyatong et al. [107]	2008	PA and C3G in purple glutinous rice bran	Dose-dependent cytotoxic effect on X63, a mouse-plasma cancer cell line of myeloma cells.

Kannan et al. [108]	2009	Peptide hydrolysates derived from rice bran	Cytotoxicity effect of <5 kDa peptide fraction separated from rice bran protein hydrolysate against HCT-116.

Kong et al. [98]	2009	Rice bran cycloartenyl ferulate	Induced apoptosis in SW480 and SW620 cells through activation of caspase-3 and caspase-8.

Kannan et al. [109]	2010	Peptides derived from defatted rice bran	Inhibited Caco-2 and HCT-116 cells growth.

Nurul-Husna et al. [110]	2010	Rice bran phytic acid	Suppressed the proliferation of HT-29 cells.

Chen et al. [63]	2012	Red rice bran	Exhibited strong inhibition on leukemia, cervical, and stomach cancer cells.

Forster et al. [71]	2013	Rice bran (variety Jasmine 85)	Exhibited a strong inhibitory effect against Caco-2 and HT-29 cells.

Takashima et al. [111]	2013	Water and ethanol extracts of rice bran	Markedly inhibited the growth of LS174T cells.

Tan et al. [72]	2013	WBR and methanol extract of brewers' rice	Inhibited the proliferation of HT-29 cell line.

C3G: cyanidin 3-glucoside; Caco-2: colon cancer cells; HCC70: human breast cancer cells; HCT-116: human colon cancer; HepG2: liver cancer; HT-29: human colorectal cancer; IC_50_: inhibition concentration for 50%; LS174T: human colon cancer; MCF-7: human breast adenocarcinoma; PA: proanthocyanidin; SW480: human colon cancer; SW620: human colon cancer; WBR: water extract of brewers' rice.

**Table 7:** Anticancer effect of rice by-products *in vivo*.

Author(s)	Year	Components	Findings
Yasukawa et al. [112]	1998	Rice bran cycloartenol ferulate	Suppressed tumor promotion in 2-stage skin carcinoma in ICR mice.

Kawabata et al. [113]	1999	Rice germ	Suppressed ACF formation and reduced incidence of colonic adenocarcinoma.

Mori et al. [114]	1999	Rice germ	Reduced the numbers of ACF/colon, ACF/cm^2^, and aberrant crypts/colon in the group treated with AOM + GABA-enriched defatted rice germ (2.5% in diet) and the group treated with AOM + rice germ (2.5% in diet) compared with that of the group treated with AOM alone. Inhibited AOM-induced large bowel neoplasms incidences in Fisher 344 rats.
		Ferulic acid and defatted rice germ	Reduced the incidence of tongue carcinomas and preneoplastic lesions in Fisher 344 rats.

Mori et al. [115]	2000	Rice germ and GABA-enriched defatted rice germ	Suppressed AOM-induced ACF and colon carcinogenesis in rats.

Cai et al. [97]	2005	Tricin from rice bran	Reduced the number of intestinal adenomas via the suppression of COX activity and inhibited PGE_2_ production.

Kim et al. [100]	2007	Methanol extract of rice husk	Decreased colonic preneoplastic ACF formation by 35%.

Verschoyle et al. [8]	2007	30% rice bran	Reduced numbers of intestinal adenomas in APC^Min^ mice.

Kawasaki et al. [116]	2008	Rice bran hemicellulose	Inhibited the total number of colon tumors and tumor incidence in Fisher 344 rats.

Boateng et al. [117]	2009	Rice bran	Dietary administration of 5% and 10% of rice bran significantly (*p* < 0.05) reduced the AOM-induced colon tumors incidence in male Fisher 344 rats after 44 weeks of feeding.

Panala et al. [118]	2009	Rice bran oil	Inhibited incidence of ACF in Fisher 344 rats.

Wang et al. [119]	2009	Defatted rice bran sulfated polysaccharide (SRBPS2a)	Suppressed implanted EMT-6 breast tumor cells growth in BALB/c mice.

Norazalina et al. [96]	2010	Rice bran phytic acid	Reduced the number of ACF in the distal, middle, and proximal colon.

Li et al. [120]	2011	Rice bran	Suppressed the number of ACF and expression of COX-2 in the middle colon.

Shih et al. [121]	2011	Rice bran oil	Suppressed colon tumor formation, mucin-depleted foci, and ACF especially sialomucin-producing ACF in 1,2-dimethylhydrazine/dextran sodium sulphate induced colitis-associated colon cancer after 13 weeks of feeding.

Kim et al. [122]	2012	Rice bran *γ*-oryzanol	Inhibited colon tumor growth in mice.

Choi et al. [123]	2013	10% (w/w) black and brown rice brans	Inhibited the colon transplanted tumors in mice.

Shafie et al. [124]	2013	Rice bran phytic acid	Markedly reduced *β*-catenin and COX-2 expression in colon tumors.

Tan et al. [55]	2014	Brewers' rice	Markedly reduced colon tumor in rats.

ACF: aberrant crypt foci; AOM: azoxymethane; APC: adenomatous polyposis coli; COX: cyclooxygenase; COX-2: cyclooxygenase-2; GABA: gamma-aminobutyric acid; PGE_2_: prostaglandin E_2_.
